# Time of insemination in relation to pregnancy rates in beef cattle after oestrus detection with automated activity monitoring system

**DOI:** 10.1186/s13028-023-00685-y

**Published:** 2023-06-09

**Authors:** Caroline Sorknes Haadem, Ingrid Hunter Holmøy, Ane Nødtvedt, Adam Dunstan Martin

**Affiliations:** Department of Production Animal Clinical Sciences, Norwegian University of Life Sciences, 8146 Dep., 0033 Oslo, Norway

**Keywords:** AI time, Heat detection, Precision farming, Progesterone, Suckler cow

## Abstract

**Background:**

The timing of artificial insemination is critical to achieve acceptable results in cattle production systems. Over the past 60 years the length and expression of oestrus in dairy cattle has altered. Recent studies have indicated the optimal timing for insemination after the commencement of oestrus may now be earlier than traditional recommendations in beef cattle, as is the case in dairy cattle. The aim of the current study was to evaluate the effect of time from onset of oestrus [as determined by an automated activity monitoring system (AAMS)] to artificial insemination (AI) on pregnancy outcome in Norwegian beef cattle. Five commercial beef suckler herds participated in a cohort study by providing data on the time of AAMS alarm and time of AI. Blood sampling on the day of AI was performed and serum progesterone concentration measured. Pregnancy detection was performed by transrectal ultrasonography and aging of the fetus performed when necessary. A mixed logistic regression model was fitted to study the effect of time from AAMS alarm to AI on pregnancy outcome. Time categories used in the model were < 12 h, 12–24 h, and > 24 h.

**Results:**

AI periods (n = 229) with serum progesterone concentration < 1 ng/mL were available for analysis. Overall pregnancy risk per AI for the whole study period was 65.5%, with an inter-herd variation from 10 to 91%. Median time elapsed from AAMS alarm to AI was 17.75 h. Herd affected pregnancy outcome (P = 0.001), while breed and parity status (heifer/cow) did not. The time category closer to AAMS alarm 0–12 h showed a numerically lower pregnancy risk as compared to the baseline group which had AI 12–24 h after onset of oestrus.

**Conclusion:**

This study found no evidence to support a change in the recommended timing of AI in beef suckler cows.

**Supplementary Information:**

The online version contains supplementary material available at 10.1186/s13028-023-00685-y.

## Background

Artificial insemination (AI) in cattle has been available for almost a century and is the dominant form of service used in intensive dairy production systems [1]. However, worldwide only 5% of beef cows are served by AI as opposed to 75% of dairy cows [1]. A similar pattern is seen in Norway where less than 15% of beef cows are bred by AI compared to 85% of the dairy cattle [2]. This difference is seen despite the potential benefits for disease control, genetic improvement and management being similar across beef and dairy production systems. Previous research has shown that time constraints, perceived inconvenience and difficulties with oestrus detection are reasons why beef farmers opt not to use AI [3]. This is despite there only being a moderate level of irregular oestrus intervals in beef cows [4]. The problem of identifying cows in oestrus can be overcome using technological devices for oestrus detection, such as automated activity monitoring systems (AAMS). Several such devices exist and have been investigated over the years, mainly in dairy herds [5, 6]. With the rapid advancement of precision farming experienced today, research efforts on in-herd sensor technology performance are warranted in all sectors of cattle farming.

The procedure of AI aims to ensure that an adequate number of capacitated motile spermatozoa are in the caudal isthmus of the oviduct at the time of ovulation to maximise the chance of fertilization [7]. Given that the capacitation reaction, travel through the uterus, lifespan and point of ovulation are all time dependent the success of AI is dependent on AI being performed at the correct time. Research directed at the optimal timing of AI in dairy cattle has generated the commonly applied “a.m./p.m. rule” [8], which suggests that cows displaying their first signs of oestrus in the morning (a.m.) should be bred in the evening (p.m.) while cows coming into heat in the evening are bred the following morning. In Norway the majority of AI is performed by veterinary surgeons or AI technicians on cows identified as being in oestrus by farmers. Synchronisation of oestrus and hormonal treatment of cattle is not common practice and therefore knowledge of oestrus behaviour relative to time of insemination and pregnancy outcome in naturally cycling cows in Norway is important [9]. Current AI practice in Norway requires a farmer to call a veterinary surgeon or technician before 9 a.m. to order an insemination for the same day. Whilst AI practitioners will often try to be helpful in the timing of insemination, the large distances in Norway mean that often cattle are inseminated outside of the classic a.m./p.m. recommendation.

The genetics, environment, disease incidence and management of cattle have changed dramatically since the a.m./p.m. rule was described in the 1940’s. Particularly over the past few decades changes in duration of oestrus, level of oestrus behaviour displayed and potentially even oestrous cycle length in dairy cattle have been reported [10, 11]. The selection, disease, management systems and production pressures on beef and dairy systems have been very different over the past century. Given this and that inter-breed differences in oestrus behaviour exist studies performed in dairy cattle should not automatically be applied to beef cattle. Previous research has reported that ovulation occurred 2.5–8.5 h closer to onset of oestrus in a herd of Hereford cows compared to the expectation for Holstein cows [12, 13].

There is no tradition for using synchronization and fixed-time AI in Norwegian cattle herds, and because there is an increase in consumer resistance toward hormonal treatment for fertility in cows, this is an unlikely strategy also in the future [14, 15]. Therefore, it is important to increase the knowledge regarding the optimal timing of AI in beef cows experiencing naturally occurring oestrus as few modern studies address this issue [16]. The optimal insemination time for beef heifers that have undergone oestrus synchronization treatments has been estimated to be 8–12 h after onset of oestrus [17]. However, there are no recent studies on optimal AI time in naturally cycling beef cows. In a recent study performed on a herd of naturally cycling Hereford cows, ovulation was found to occur 23 h after onset of oestrus as defined by an AAMS alarm. In this herd 25% of cows and heifers had ovulated within 19 and 11 h, respectively [13]. However, risk of pregnancy to AI, the outcome of interest to the farmer, was not studied.

This aim of the present study was therefore to add to the knowledge base AI timing in beef cattle production systems by testing the hypothesis that beef cows inseminated closer to AAMS alarm would have an increased risk of pregnancy when compared to cows inseminated later relative to the time of AAMS alarm. Thus, the objective of this study was to evaluate the effect of time from AAMS alarm to AI on pregnancy outcome in Norwegian beef cattle.

## Methods

### Study population

This prospective cohort study recruited commercial beef suckler herds in Norway which used the AAMS Heatime® (SCR Engineers Ltd., Israel) for oestrus detection to facilitate AI. A list of herds that had purchased the Heatime® system was obtained by contacting the distributor of Heatime® in Norway, Geno SA (Hamar, Norway) and recruitment to the study was based on this list. Herds with more than 20 recorded beef cattle inseminations per year were contacted by phone and followed up by text message or mail if they did not reply. If the farmer did not answer the phone after four attempts on different days, and did not reply to messages or mail, the farm was excluded. Due to a low interest to participate in our study, we recruited additional herds by convenience and by publishing in a journal for farmers. Therefore, all farms willing to participate in the study were included regardless of number of inseminations they had had in the year before the study. Only herds where veterinarians performed AI were recruited to participate in the study.

### Detection of oestrus and insemination

The oestrous cycle of the heifers and cows included in this study was natural, i.e., no hormonal treatments or oestrus synchronization programmes were used. Individual cow activity was measured using a proprietary movement sensor included in the Heatime® neck collar, which record all cow movement and activity intensity as well as details on rumen activity (www.scrdairy.com/cow-intelligence/technology.html), continuously. These data were transferred from the neck collar to the central computer in 2-hour blocks by radio transmission. Activity measurements recorded in the central computer were used to establish the threshold of oestrus activity according to the manufacturer’s guidelines (SCR Engineers Ltd.). Oestrus was defined as beginning at the time of the AAMS alarm. Upon the detection of oestrus an alarm is sent by email or text message to the farmer indicating the animal is likely to be in oestrus. All inseminations in this study were performed after oestrus was identified by the AAMS. Animals that the AAMS did not recognised as being in oestrus, but that were judged by the farmer to be in oestrus and inseminated, were excluded from analysis.

The farmers recorded the date and time for each heifer and cow when the AAMS gave the alarm and onset of oestrus was defined as this time point on a data-entry sheet designed for this study. The data entry points were cross checked with the data available on the AAMS system for accuracy. The herd’s practicing veterinary surgeons performed the AI in the usual manner. All the veterinary surgeons have passed an additional postgraduate course in cattle AI required by Geno SA. The farmer or veterinary surgeon noted the time and date of the AI, and the bull used. Animals with more than one AI within a period of 48 h (dual inseminations) were excluded from analysis.

### Blood sampling and analysis

The veterinary surgeon who performed the AI took a blood sample from the coccygeal or jugular vein (veterinarian’s preference) of the inseminated animals immediately after AI was completed. Samples were collected with a Vacutainer system (Venoject 0.9 × 40 mm, Terumo Europe N.V., Leuven, Belgium), into a 4.0 mL Vacuette, Z serum cloth activator (Greiner Bio-One International GmbH). The blood samples were centrifuged at 3000 x *g* for 10 min at 20° C by the veterinary surgeon who took the sample on the day the sample was taken. The serum was transferred to 2.0 mL Eppendorf™ microtubes with a single-use pipette and stored in a freezer holding − 20° C. The serum samples were analysed in duplicate for progesterone concentration by commercial ELISA at Ridgeway Research Ltd., UK. The laboratory reported the intra-assay coefficient of variation to be 9.6% and the inter-assay coefficient variation to 6.6%. Serum samples which had a progesterone concentration greater than 1 ng/mL serum were defined as belonging to a cow in the luteal phase, and therefore not in oestrus [13].

### Outcome and pregnancy detection

Detection of pregnancy was performed by transrectal ultrasonography (Easi-Scan, IMV imaging Ltd., Scotland), by a diplomate of the European College of Animal Reproduction between 28 and 119 days after AI. Pregnancy risk was defined as the number of pregnant cows/number of AIs. Pregnancy detection was performed after the conclusion of the AI period prior to the introduction of the clean-up-bull in the herds wherever possible. In the herds where this was not possible, pregnancy detection was performed after the grazing period. In herds using a natural mating after the insemination period had finished, aging of the fetus was done at pregnancy exam to determine whether the heifer/cow was pregnant by the AI or the bull. The time span from the last AI to the introduction of a bull was no less than three weeks for all animals included in the study. Fetal aging was also performed if a cow had several oestrus events with a recorded AI. Age determination was performed measuring crown-rump length (CRL) of the fetuses aged 6–10 weeks [18], or trunk diameter (TD) in fetuses aged 11–17 weeks. Cases where fetal age could not be estimated by CRL, or TD were excluded.

The binominal outcome of interest was pregnancy after each AI. The unit of observation was AI following AAMS detected oestrus. Therefore, repeated inseminations in the same animal on separate oestrus events were possible and included as independent observations.

### Explanatory and extraneous variables and statistical analysis

The main explanatory variable was the time elapsed from the beginning of oestrus (AAMS alarm) until AI. Data were managed and cleaned in Excel before transfer to Stata (Stata SE/15, Stata Corp., USA) for statistical analysis. Linearity between risk of pregnancy and time from AAMS alarm to AI was assessed for using the command lintrend. A mixed logistic regression model was used to study the effect of time from AAMS alarm to AI on pregnancy outcome. Explanatory variables were identified through a causal diagram and evaluated by monitoring changes in the estimates of the multivariable model when the factor was included in/excluded from the model. The explanatory variables considered as possible were herd, breed of cow, parity (heifer/cow) and the use of different semen extenders and bull. The model was built by a manual forwards selection procedure to control for the possible confounders. Time from AAMS alarm to AI was divided into three groups for the mixed logistic regression model. The explanatory variable of time from AAMS alarm to AI was categorized into three intervals (< 12 h, 12–24 h and > 24 h).

## Results

### Study sample

The number of eligible herds and those included in the study, along with the number and reasons for exclusion, are presented as Additional file 1: Fig. S1 in the additional file accompanying this paper. All inseminations from the herds, as well as number and reasons for exclusion from further analysis are detailed in Additional file 1: Fig. S2 of the additional file accompanying this paper. Of the herds included in the study all except one was managed in free-stall indoor system during the insemination period before being pasture grazed after. Four herds used natural mating after the period of AI. The study herds were situated in the central and south-western regions of Norway.

The final study dataset included 229 inseminations performed in 159 cows from 5 herds between the end of March 2017 until the middle of July 2019. Length of the insemination periods in each herd ranged from two to five months. Each herd contributed between 11 and 90 inseminations. Table 1 shows the distribution of inseminations between farms, number of cows included in the study for each farm as well as the farms’ location in Norway. Fifty-three different bulls were used. Cow breeds included observations from Charolais, Hereford, Aberdeen Angus, Limousin, Blonde d’Aquitaine, and beef crossbreeds. Conventional semen extenders were used in 221 inseminations and the Spermvital® extender (www.spermvital.com) was used in 8 inseminations.

**Table 1:** Distribution of individuals and inseminations by farm, nulliparous or pluriparous and timing of AI relative to oestrus alarm from an automated activity monitoring system

Herd*	Number of		Heifer insemination timing			Cow insemination timing			Total number of inseminations	
	Heifers	Cows	< 12 h	12–24 h	> 24 h	< 12 h	12–24 h	> 24 h	Heifers	Cows
1	29	51	1	17	14	8	31	9	32	58
2	9	19	4	8	1	13	20	12	13	45
3	12	33	2	11	1	8	33	5	14	46
4	0	7	0	0	0	6	3	1	0	10
5	11	0	4	5	2	0	0	0	11	0
Total			11	41	18	35	87	27	70	149

^*^Herds 1 and 5 are located in central Norway. Herds 2, 3 and 4 are located in south-eastern Norway

### Descriptive statistics

The number of inseminations and risk of pregnancy following AI for each of the AI time groups are listed in Table 2. The overall pregnancy risk of animals included in the study was 0.66 (150/229). Table 3 lists number of inseminations and pregnancy risk for the variables herd, breed and parity. Median time of AI from onset of oestrus was 17.75 h and 90% of inseminations were performed within 27 h of the oestrus detection alert (range: 0–40 h). Only the herd variable effected pregnancy risk (P = 0.002) based on univariable analyses.

**Table 2:** Inseminations and pregnancy risk for the explanatory variable time from automated activity monitoring system oestrus alarm to AI

Time from AAMS alarm	Number of inseminations	Pregnant (%)
< 12	46	25 (54.3)
12–24	128	87 (68.0)
> 24	55	38 (69.1)
Total	229	150 (65.5)

**Table 3:** Distribution of inseminations by specified explanatory variables

Variable	Number of inseminations	Pregnant (%)*
Parity		
Heifer	70	50 (71.4)
Cow	159	100 (62.9)
Herd		
1	90	61 (67.8)
2	58	34 (58.6)
3	60	44 (73.3)
4	10	1 (10)
5	11	10 (90.9)
Breed of cow		
Charolais	65	39 (60.0)
Blonde d’Aquitaine	60	44 (73.3)
Hereford	21	11 (52.4)
Aberdeen Angus	22	15 (68.2)
Limousin	6	5 (83.3)
Beef crossbreed	55	36 (65.5)
Total	229	150 (65.5)

*Pregnancy risk (%) provided for each variable level

### Odds of pregnancy

There was no observed difference in pregnancy risk for animals in the different groups defined by time from AAMS alarm (< 12 h, 12–24 h, and > 24 h). Compared to the baseline of 12–24 h after onset of oestrus, cows inseminated between 0 and 12 h after onset of oestrus had a lower risk of becoming pregnant (OR 0.71), however the difference was non-significant. The possible explanatory variables considered included: parity (heifer/cow), and cow breed were considered possible explanatory variables but did not affect the estimates and were omitted from the final model. Similarly, bull was tested as a random effect in the model, but the effect of bull was non-significant and therefore not included in the model. Of the possible explanatory variables, only herd was kept in the final model. It led to a slight change in the estimated effect on pregnancy risk of time from AAMS alarm to AI. Estimates from the regression model are presented in Table 4.

**Table 4:** Odds ratios (OR) and 95% confidence intervals (CI) for pregnancy risk by time and farm

	OR	95% CI	P
Time to AI ^*^			
<12 h	0.71	0.33–1.51	0.38
12–24 h	Baseline		
>24 h	1.13	0.55–2.32	0.74
Farm			
1	Baseline		
2	0.73	0.36–1.48	0.38
3	1.38	0.66–2.92	0.39
4	0.06	0.01–0.54	0.01
5	5.39	0.65–44.94	0.12

*Time from automatic activity monitoring system alarm of oestrus until articicial insemination

## Discussion

This study found no evidence to support the hypothesis that beef cows inseminated less than 12 h after oestrus as determined by a commercially available AAMS system would be more likely to get pregnant than those inseminated more than 12 h after the AAMS detected oestrus. This contradicts some previous studies in dairy cattle which have observed an increased risk of pregnancy when AI was performed within 12 h of activity threshold [19, 20]. It also contradicts a previous study on ovulation time in a Hereford herd performed by our group which showed as many as 25% of heifers and cows ovulated between 11 and 19 h after AAMS alarm, respectively [13]. These ‘early ovulating’ animals would hypothetically not have been able to become pregnant if inseminated according to the a.m./p.m. rule, because there would have been insufficient time for spermatozoa to be transported to the oviduct and undergo capacitation. Further, animals with a short insemination to ovulation interval (< 12 h), are more likely to develop embryos of poor quality, as time of ovum fertilization affect early embryo development [21]. However, in the current study we observed a marginally lower risk of pregnancy in animals inseminated < 12 h after onset of oestrus, but the effect was not statistically significant. As we did not palpate the ovaries transrectally before or after AI in our study, we have no record on time of ovulation. The only factor to significantly affect the risk of pregnancy in the current analysis was herd.

The Heatime® device notified the farmer once cow activity increase above a set threshold. This threshold shows inter-cow variation as the length of oestrus but also the intensity and duration of oestrus display differs between animals. Inter-cow difference in oestrus expression is explained by genetics [22, 23], environmental- and managemental factors (such as flooring, stocking density, herd size, climate), which differ among herds [24–29]. In addition, time elapsed from calving will influence fertility in individual cows. Herd management practices, especially oestrus detection, were found to have a greater effect on reproductive performance than intrinsic cow characteristics in a study aiming to quantify factors affecting productivity in dairy herds [30]. It is also important to realize that other managemental practices such as the veterinarian performing AI, the handling facilities available and time of separation of one cow from the rest of the herd (while waiting to be inseminated), are unique to each farm and will all affect fertility. The variation in fertility by herd is generally not accounted for in studies like the present one, as available research about timing of AI on pregnancy risk in beef and dairy cattle tend to take place on a single farm or research facility.

Breed affects the duration of oestrus and thus timing of ovulation relative to the onset of oestrus behaviour [11, 19, 23, 31]. However, our results did not imply any effect of breed on pregnancy risk, further emphasizing the importance of herd. To correctly measure the effect of breed it is necessary to include herds housing several breeds as this variable frequently correlates with herd. The animals in the study of ovulation time in a Hereford herd, on which we based our hypothesis, may have been extraordinary in terms of early ovulation or increase in activity late in oestrus, thus decreasing time to ovulation from AAMS alarm [13]. The contradictory nature of our study to the study by Nelson et al. [13], could therefore in part be explained by different conditions in housing and management between farms.

This study was performed as a prospective cohort study [32]. To minimize misclassification of outcome, a specialist in theriogenology performed all the pregnancy diagnoses. False negatives could have occurred in the case of embryonic death pre-pregnancy diagnosis. Our outcome (pregnancy as defined by transrectal ultrasonographic examination after day 28), is a relevant measure of fertility. Although studies on time of ovulation are of value in relation to timing of AI, the industry standard of reproductive efficiency is pregnancy or calving.

To ensure comparable risk of pregnancy, all included animals had low serum progesterone (< 1 ng/mL) at the time of AI. This cut off was set to excluding pregnant animals or animals in dioestrus that might have been a part of a sexually active group [33]. However, whilst all blood samples in this study underwent centrifugation and freezing on the day of the study the time from sampling to processing was not recorded. The concentration of progesterone in full bovine blood has been shown to decline overtime [34], and therefore it is possible that some pregnant cows or cows in dioestrus could have been misclassified as having low progesterone concentrations, and therefore being in oestrus, when they were not, although the authors feel the number of animals potentially involved would have been very small. Further low serum progesterone concentrations are not diagnostic for oestrus and the possibility that some cows were in anoestrus at the time of insemination cannot be excluded.

Risk of pregnancy could be influenced by inseminator competence. To minimize the effect this could have on the results all inseminations were performed by veterinarians who had completed an additional training course provided by the breeding organization Geno. The veterinarian’s performance is evaluated by the breeding organization (Geno) who instigates mandatory training if performance falls below expected levels, thus the effect of inseminator bias should be minimized.

In this study, misclassification of exposure was minimized by excluding animals inseminated solely based on observation of oestrus. A lack of independence between inseminations from the same bull because individual bulls have different fertility exists in this study [35]. This was accounted for in the model building process by including bull as a random effect in the model. However, the random effect of bull hardly altered the odds ratio of the predictor variables and statistically the effect was not significant (P = 0.21). Therefore, to preserve the principles of parsimony bull was excluded from the model. The spatial clustering of cows within herds was accounted for by including herd as a fixed effect in the multivariable model.

An important point of the present study was to include several farms. There are 94,780 registered beef suckler cows in Norway, and only 13.8% of these are bred (at least once) by AI [36]. The source population in our study differs from the general population of Norwegian beef cattle in terms of breeding strategies (AI vs. natural mating). Thus, the results are not necessarily applicable to Norwegian beef cows in general and external validity of our study might be low. Our source population consisted of herds where the farmers had invested in AAMS and used AI to increase productivity. Therefore, we assume these herds to be more homogenous in terms of management system compared to the average beef suckler herd in Norway. However, herds with less than 20 cows bred by AI the year prior to the study were not contacted, thus our study sample may be skewed towards the larger herds. All studies relying on farmer’s willingness to participate will be subjected to selection bias. Due to a low interest to participate in our study, we recruited additional herds by convenience and by publishing in a journal for farmers. In addition, the final study sample included no farms from the northern or southern parts of the country, as the few herds located here and meeting the inclusion criteria were unwilling to participate.

Whilst this study could be criticized for its relatively low statistical power, the challenges presented by performing a study of this nature precluded a larger study population. Field studies are notoriously challenging, particularly those taking place over large geographical distances. To illustrate this whilst performing this study three of the farms dropped out. Despite these limitations, we feel confident the study has merit as it describes the current situation in Norway regarding insemination in beef suckler herds. Furthermore, it finds the current recommendations to be at least equal to a hypothesis driven alterative practice of earlier AI in beef suckler herds.

## Conclusion

This study found no evidence to support a change in the current recommendations regarding the timing of AI in beef cattle. The current practice in Norway is that a farmer calls a veterinarian before 9 a.m. to have their cows inseminated during the same day; continuation of this practice is supported by the findings of this study. The effect of time of AI after onset of oestrus on pregnancy outcome in beef cattle highly depends on herd and not necessarily breed. Thus, measures should be taken to ascertain that the herdsman understands oestrus behaviour and display in his herd as well as its effect on AAMS alarm.

## Supplementary Information

**Additional file 1: Fig. S1.** Flow chart of eligiblestudy herds. **Fig. S2.** Flow chart showingincluded and excluded observations/inseminations and reasons from exclusionfrom the study.

## Data Availability

The datasets used for the current study are available from the corresponding author on reasonable request.
