# Supplementary material for: Time of insemination in relation to pregnancy rates in beef cattle after oestrus detection with automated activity monitoring system
Source: Acta Vet Scand. 2023 Jun 9;65:20. doi: 10.1186/s13028-023-00685-y (PMC10257325; doi:10.1186/s13028-023-00685-y)

**Accompanying file; ‘Time of insemination in relation to pregnancy rates in beef cattle after oestrus detection with automated activity monitoring system’** *Acta Veterinaria Scandinavia* (2023) by Haadem *et al*.

**Figure S1**. Flow chart of eligible study herds.


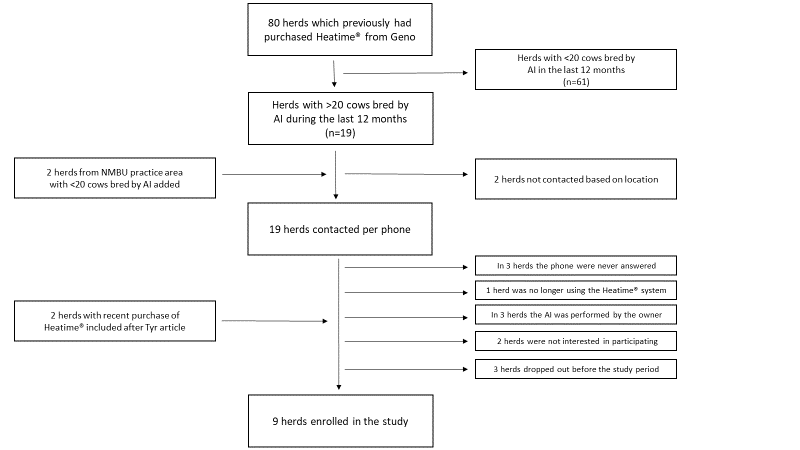


NMBU = Norwegian University of Life Sciences

**Figure S2.** Flow chart showing included and excluded observations/inseminations and reasons from exclusion from the study.


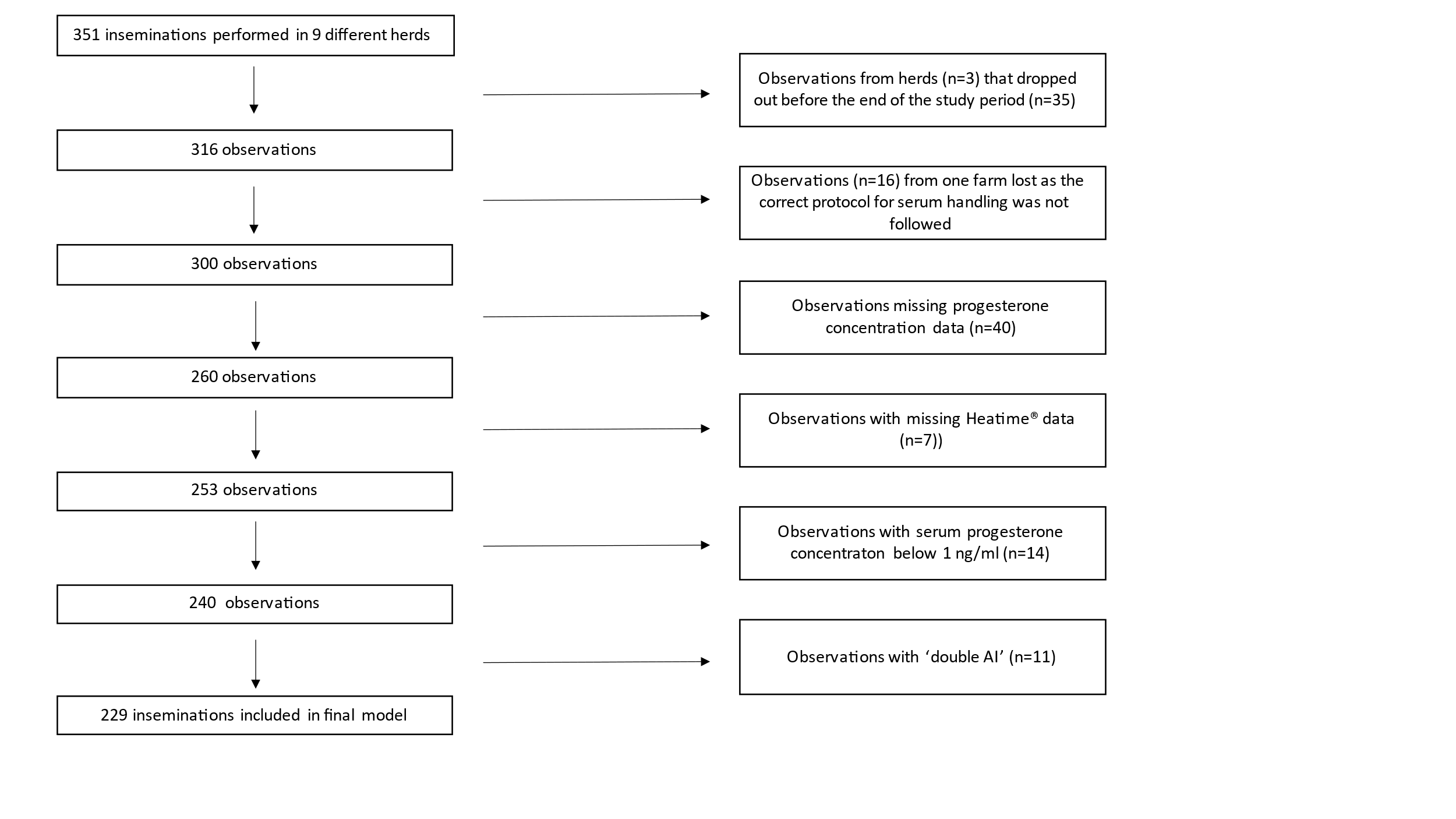

Supplement: Supplementary file 1 — Additional file 1: Fig. S1. Flow chart of eligiblestudy herds. Fig. S2. Flow chart showingincluded and excluded observations/inseminations and reasons from exclusionfrom the study. [file 13028_2023_685_MOESM1_ESM.docx]
